# Road Lane Detection Robust to Shadows Based on a Fuzzy System Using a Visible Light Camera Sensor

**DOI:** 10.3390/s17112475

**Published:** 2017-10-28

**Authors:** Toan Minh Hoang, Na Rae Baek, Se Woon Cho, Ki Wan Kim, Kang Ryoung Park

**Affiliations:** Division of Electronics and Electrical Engineering, Dongguk University, 30 Pildong-ro 1-gil, Jung-gu, Seoul 100-715, Korea; hoangminhtoan@dongguk.edu (T.M.H.); qorskfo1023@hanmail.net (N.R.B.); jsu319@naver.com (S.W.C.); yawara18@dongguk.edu (K.W.K.)

**Keywords:** road lane detection, shadows, fuzzy system, line segment detector

## Abstract

Recently, autonomous vehicles, particularly self-driving cars, have received significant attention owing to rapid advancements in sensor and computation technologies. In addition to traffic sign recognition, road lane detection is one of the most important factors used in lane departure warning systems and autonomous vehicles for maintaining the safety of semi-autonomous and fully autonomous systems. Unlike traffic signs, road lanes are easily damaged by both internal and external factors such as road quality, occlusion (traffic on the road), weather conditions, and illumination (shadows from objects such as cars, trees, and buildings). Obtaining clear road lane markings for recognition processing is a difficult challenge. Therefore, we propose a method to overcome various illumination problems, particularly severe shadows, by using fuzzy system and line segment detector algorithms to obtain better results for detecting road lanes by a visible light camera sensor. Experimental results from three open databases, Caltech dataset, Santiago Lanes dataset (SLD), and Road Marking dataset, showed that our method outperformed conventional lane detection methods.

## 1. Introduction

Detecting road lane markings is an important task in autonomous vehicles [1,2,3]. Most recent algorithms for lane detection are vision-based. Images captured from various types of cameras such as visible light camera sensors are processed to extract all meaningful feature data such as edges, lane orientation, and line boundaries, and they are combined with the distance information measured by radar sensors. A vision-based system requires camera calibration before operating, good environmental situations and road conditions, and high processing speed to detect lane boundaries in real time to match the speed of the vehicles. Therefore, most of the methods based on handcrafted features propose three main steps of processing [1,4,5,6,7]: (1) pre-processing: enhancing illumination of the original image captured from the camera; (2) main-processing: extracting features of road lane markings such as edges, texture, and color; and (3) post-processing: removing outliers or clustering detected line segments.

Unlike traffic signs, severe shadows can exist on road lanes, and this factor leads to challenging problems for automatic recognition and classification of road lanes. For example, owing to the effect of overly bright or overly dark illuminations, a solid lane can be divided into smaller units; therefore, it can be falsely recognized as a dashed lane [6]. Therefore, we propose a method of road lane detection by using a fuzzy inference system (FIS) to overcome the effect of shadows on input images. Detailed explanations of previous approaches are provided in the Section 2.

## 2. Related Works

Previous research on road lane detection used visible light and night-vision cameras, or combinations of the two, to enhance the accuracy. Previous studies on camera-based lane detection can be classified into model-based and feature-based methods. The first approach uses the structure of the road to create a mathematical model to detect and track road lane named model-based methods. A popular mathematical model is B-splines [4,8,9,10,11]; this model can form any arbitrary shape using a set of control points. Xu et al. detected road lanes based on an open uniform B-spline curve model and maximum deviation of position shift (MDPS) method to search control points, but the method resulted in a large deviation, and, consequently, it could not fit the road model for the case when the road surface was not level [8]. Li et al. adopted an extended Kalman filter with a B-spline curves model for continuous lane detection [9]. Truong et al. [4] combined the vector-lane-concept and non-uniform B-splines (NUBS) interpolation method to construct the left and right boundaries of road lanes. On the other hand, Jung et al. used the linear model to fit the near vision field, while the parabolic model was used to fit the far field to approximate lane boundaries in video sequences [12]. Zhou et al. presented a lane detection algorithm based on a geometrical model and the Gabor filter [13]. However, they assumed the road in front of the vehicle was approximately planar and marked, which is often correct on the highway and freeway; and the geometrical model built in this research required four parameters: starting position, lane orientation, lane width, and lane curvature. In previous research [14], Yoo et al. proposed a lane detection method based on gradient-enhancing conversion to guarantee an illuminating-robust performance. In addition, an adaptive Canny edge detector, a Hough transformation (HT), and a quadratic curve model are used in their method. Li et al. adopted an inverse perspective mapping (IPM) model to locate a straight line in an image [15]. The IPM model was also used in [5,15,16,17,18]. Chiu et al. proposed a lane detection method based on color segmentation, thresholding, and fitting the model of a quadratic function [19].

These methods start with the hypothesis of the road model, and then match the edge with the road structure model. They only use a few parameters to model the road structure. Therefore, the performance of lane marking detection is affected by the accurate definition of mathematical model, and the key problem is how to choose and fit the road model. That is why these methods work well only when they are fed with complete initial parameters of the camera or the structure of the road.

As the second category, feature-based methods or handcrafted feature-based methods have been researched to address this issue. These methods extract features such as edges, gradient, histogram and frequency domain features to locate lane markings [6,20,21,22,23,24,25,26,27]. The main advantages are that this approach is not sensitive to the structure of road, model, or camera parameters. However, these feature-based methods require a noticeable color contrast between lane markings and road surface, as well as good illumination conditions. Therefore, some works perform a variety of color-space transformations to hue, saturation, and lightness (HSL), and luminance, chroma blue, and chroma red (YCbCr) to address this issue. In addition, others use the original red, green, and blue (RGB) image. In previous research, Wang et al. [25] combined the self-clustering algorithm (SCA), fuzzy C-mean, and fuzzy rules to enhance lane boundary information and to make it suitable for various light conditions. At the beginning of their process, they converted the RGB image into that in YCbCr space so that the illumination component can be maintained, because they only required monochromatic information of each frame for processing. Sun et al. [28] introduced the method that converts the RGB image into that in the HSI color model, and applied fuzzy C-mean for intensity difference segmentation. These methods worked well when road and lane markings produced separate clusters; however, the intensity values of the road surface and road lanes are often classified into the same cluster, and, consequently, the fundamental issue of the color lane and road lanes being converted into the same value is not resolved. Although it belongs to the model-based approach, a linear discriminant analysis (LDA)-based gradient-enhancing method was introduced in the research of Yoo et al. [14] to dynamically generate a conversion vector that can be adapted for range illumination and different road conditions. Next, they achieved optimal RGB weights that maximize gradients at lane boundaries. However, their conversion method cannot work well in a case of extremely different multi-illumination conditions. This is because they assumed that multiple illuminations are not included in one scene. Wang et al. [18] simply used the Canny edge detector and HT to obtain the line data, then created the filter conditions according to the vanishing point and other location features. First, their algorithm saved the detected lane and vanishing points in near history, then clustered and integrated to determine the detection output based on the historical data; and finally, a new vanishing point was updated for the next circuit. Convolutional neural network (CNN)-based lane detection with the image captured by camera (laterally-mounted camera) at the side mirror of the vehicle was proposed [22]. In previous research [6], the authors proposed a method for road lane detection that distinguishes between dashed and solid lanes. However, they used the predetermined region-of-interest (ROI) without the detection of the vanishing point, and used the line segment detector whose parameters were not adaptively changed according to the shadows on the road image. Therefore, their performances of road lane detection were affected by the shadows on the images.

As previously mentioned, these feature-based methods or handcrafted features-based methods work well only under visible and clear road conditions where the road lane markings can be easily separated from the ground by enhancing the contrast and brightness of the image. However, they have the limitations of detecting correct road lane in case of severe shadows from objects, trees or buildings. To address this issue, we propose a method to overcome poor illumination problems to get better results of detecting a road lane. In the following four ways, our research is novel compared to previous research.

- First, to evaluate the level of shadows in the ROI of the road image, we use two features as the inputs for FIS: hue, saturation, and value (HSV) color difference based on local background area (feature 1) and gray difference based on global background area (feature 2). Two features from different color and gray space are used for FIS to consider the characteristics of shadow in various color and gray spaces.
- Second, using FIS based on these two features, we can estimate the level of shadows depending on the output of FIS after the defuzzification process. We modeled the input membership functions based on the training data of two features and maximum entropy criterion to enhance the accuracy of FIS. The procedure of intensive training which is required in training-based method such as neural network, support vector machine, and deep learning is not necessary for using FIS.
- Third, by adaptively changing the parameters of the line segment detector (LSD) and CannyLines detector algorithms based on the output of FIS, more accurate line detection can be possible based on the fusion of the detection results by LSD and CannyLines detector algorithms, irrespective of severe shadows on the road image.
- Previous researches did not discriminate the solid and dashed lanes in the detected road lanes although it is necessary for autonomous vehicle. However, even the solid and dashed lanes are discriminated (including the detection of starting and ending positions of dashed lanes) in the detected road lanes by our method.

In Table 1, we show the summarized comparisons of the proposed and existing methods.

The remainder of this paper is organized as follows: in Section 3, our proposed system and methodology are introduced. In Section 4, the experimental setup is explained and the results are presented. Section 5 presents both our conclusions and discussions on ideas for future work.

## 3. Proposed Method

### 3.1. Overview of Proposed Method

**Figure 1:**
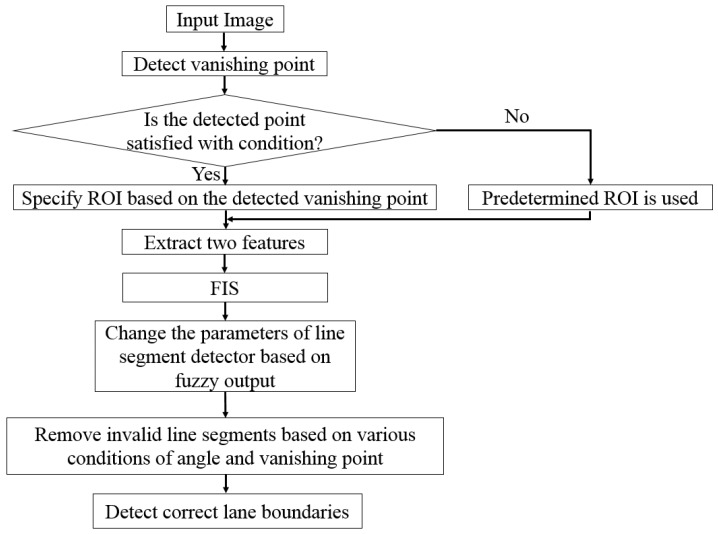
Overall procedure for the proposed method.

Figure 1 depicts the overall procedure for our method. The input image is captured by the frontal-viewing camera, and has various sizes (640 × 480 pixels or 800 × 600 pixels). In order to reduce computational complexity as well as noise, ROI for lane detection is automatically defined based on the detected vanishing point from the input image only in case that the correct vanishing point is detected (see the condition of Figure 1 in Section 3.2). If it fails to detect the correct vanishing point, the predetermined ROI is empirically defined. Next, by using two input features such as HSV color difference based on local background area (feature 1) and gray difference based on global background area (feature 2), FIS outputs the level of shadow in the current selected ROI image. Based on the FIS output value, the parameters for line segment detector algorithms are changed adaptively to enhance the accuracy of line detection. Next, three steps focus on eliminating invalid line segments based on the properties of road lanes, such as angle and vanishing point, and the correct left and right boundaries of road lanes are finally detected. We detail each step in the next sections.

### 3.2. Detect Vanishing Point and Specify ROI

**Figure 2:**
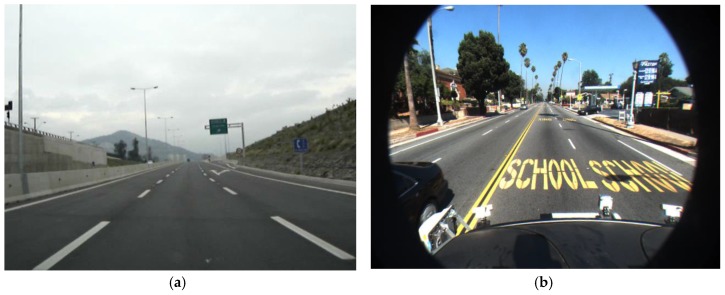

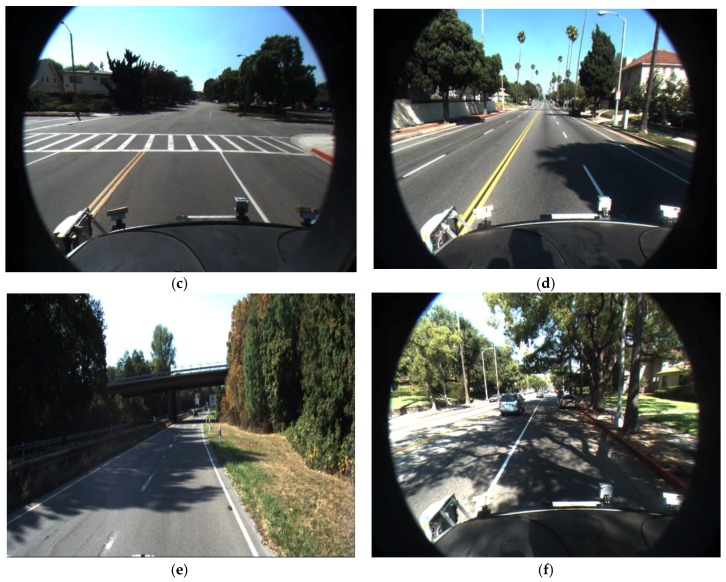
Examples of input images: (**a**) Image only with road lanes; (**b**,**c**) Images with other road markings; (**d**,**e**,**f**) Images with shadows.

In the first step, the vanishing point is detected and the ROI where the road lane is detected is automatically defined in the input image only in case that the correct vanishing point is detected. If it is failed to detect the correct vanishing point, the ROI is empirically defined. By performing the road lane detection within the ROI instead of the whole image, various noises in the captured image by the frontal-viewing camera as shown in Figure 2, can be reduced in the procedure of lane detection. In addition, the effect of environmental conditions such as sunshine, rain, or extreme weather conditions can be lessened in the case using ROI compared to that using the whole image.

In general, the vanishing point is considered one of the most important keys to retaining a valid road lane, because road lanes are assumed to converge at one point within the captured image. As shown in Figure 2, lane markings always appear within the lower part of the image, but this depends on each camera configuration, and the input image can also include other objects (e.g., car hoods in Figure 2b–f).

The vanishing point is detected as follows [24]: Left and right road lane markings usually appear like two sides of a trapezoid based on the perspective projection of the frontal-viewing camera. Therefore, we can assume that all left and right lane boundaries can converge at one point called the vanishing point. First, line segments are detected by algorithms called LSD [32,33] and CannyLines [34] using consistent texture orientation. Let *S* = $\left\{s_{1},\;s_{2},\;\ldots ,\;s_{k}\right\}$ be the set of line segments extracted from image. Each line segment $s_{i},\left(i=1,\;2,\;\ldots ,k\right)$ is defined as:

(1) $$s_{i}=\left\{x_{1i},\;y_{1i},\;x_{2i},\;y_{2i},\theta_{i}\right\},\;\left(i=1,\;2,\;\ldots ,k\right)$$

where $\left(x_{1i},\;y_{1i}\right)$ and $\left(x_{2i},\;y_{2i}\right)$ are the coordinates of the starting point and the ending point of line segment $s_{i}$, respectively. $\theta_{i}$ is the angle of line segment $s_{i}$. Next, we define the length of line segment *i*th ($len_{i}$) as the length weight ($W_{L}$). The longer line segment represents more pixels in the same direction, as well as a higher voting weight which increases the voting score. Second, Gaussian weight is calculated in Equation (2) [24]. In the voting space image, we not only consider the intersected point between two line segments, but also its 5 × 5 neighboring points. Based on Gaussian distribution, those involved points have different values to make the lines vote more smoothly, and thus improve the accuracy of the detection of the vanishing point:

(2) $$W_{G}\left(x,y\right)=\exp\left(\frac{x^{2}+y^{2}}{-2\sigma^{2}}\right)$$

where the candidate vanishing point (x,y) is computed in the neighborhood space 5 × 5 matrix, −2≤x, y≤2, σ=1.5. In Equation (2), (*x*, *y*) is the candidate vanishing point. Because there can be errors in the detected (*x*, *y*) position just based on line segments, the neighborhood space of 5 × 5 pixels based on the (*x*, *y*) is also considered by using Gaussian distribution. By using the weight of Gaussian distribution, the less weight is assigned to the position (of candidate vanishing point) far from (*x*, *y*) when determining the final vanishing point as shown in Equation (3). In addition, the less weight is given to the position (of candidate vanishing point) which is determined based on shorter line segment ($W_{L}$) as shown in Equation (3). The score of the current selected pixel is then calculated as follows:

(3) $$I{\left(x,y\right)}_{score}=W_{L}+W_{G}\left(x,y\right)$$

Finally, we create a matrix space which is the same size as the input image and initialized to 0. Next, we update the score of each element in the matrix that corresponds to each pixel in the input image by adding $I{\left(x,y\right)}_{score}$ into the current value at the same position. Here, (x,y) is coordinate of current element in matrix and it is also a coordinate of current selected pixel in input image. The point that has the largest value is considered the vanishing point [24].

**Figure 3:**
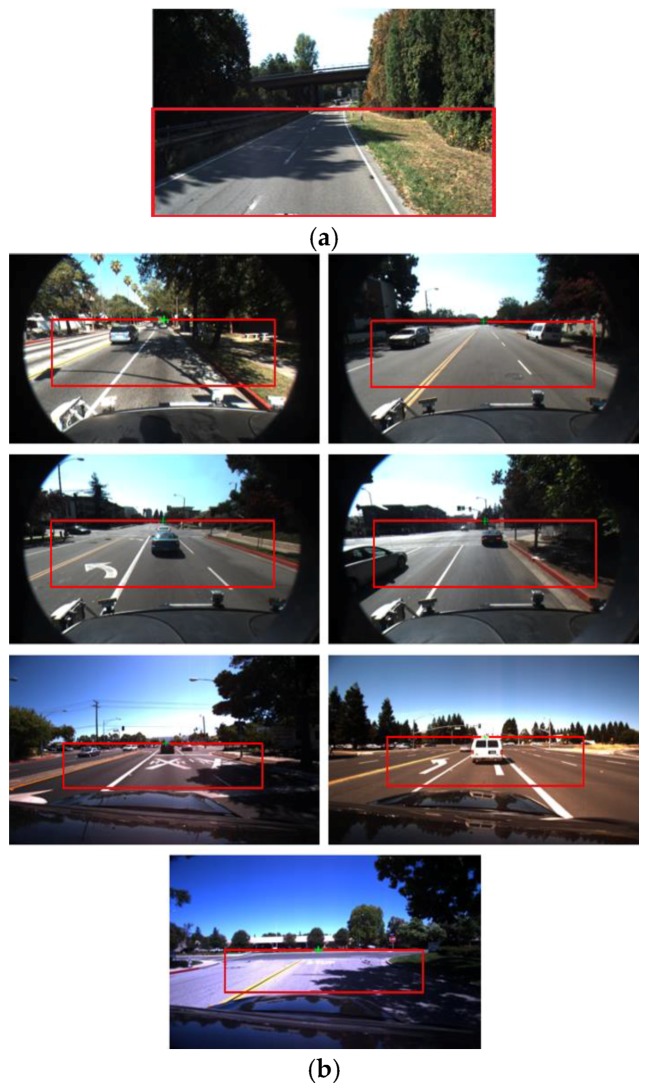
Predetermined ROI, and automatically defined ROI based on vanishing point within the input image: (**a**) Predetermined ROI; (**b**) Automatically defined RO with the detected vanishing point of green cross shape.

Figure 3b shows examples of detecting the vanishing point and defined ROI based on the vanishing point. Incorrect vanishing point caused by the car hood can be removed, and correct one is obtained, which produce the correct ROI as shown in Figure 3b. In addition, although incorrect line segments can be generated by shadows, the voting methods considering the Gaussian function-based weight and the length weight of line segment as shown in Equations (2) and (3) can prevent the detection of incorrect vanishing point by the incorrect line segments by shadows as shown in Figure 3b.

**Figure 4:**
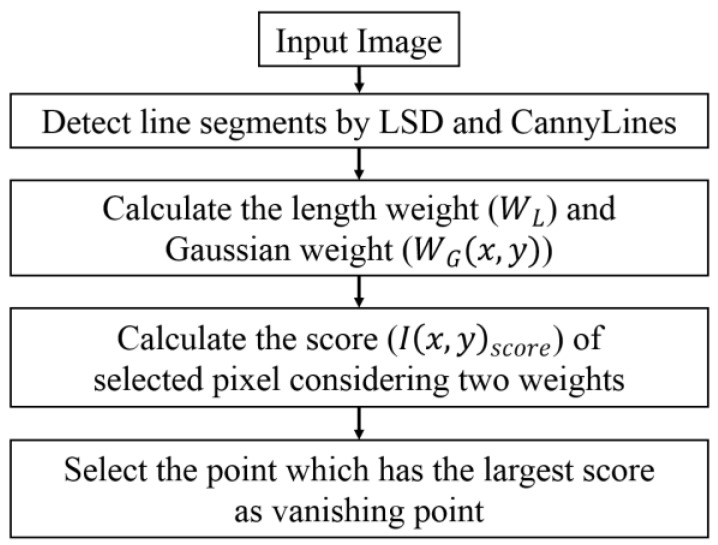
Overall procedure of detecting vanishing point.

In order to prevent an incorrect ROI caused by inaccurate detection of the vanishing point, the *y* position of the vanishing point is compared to the upper *y* position of the predetermined ROI of Figure 3a (which is manually determined according to the database). If the difference between these two *y* positions is larger than the threshold (30 pixels), the predetermined ROI is used for lane detection, assuming that detection of the vanishing point fails. The diagram of these procedures are shown in Figure 4. In next Section 3.3 and Section 3.4, we would explain the method of extracting features 1 and 2 as the inputs to FIS to measure the level of shadows.

### 3.3. Calculating Feature 1 (HSV Color Difference Based on Local Background Area)

**Figure 5:**
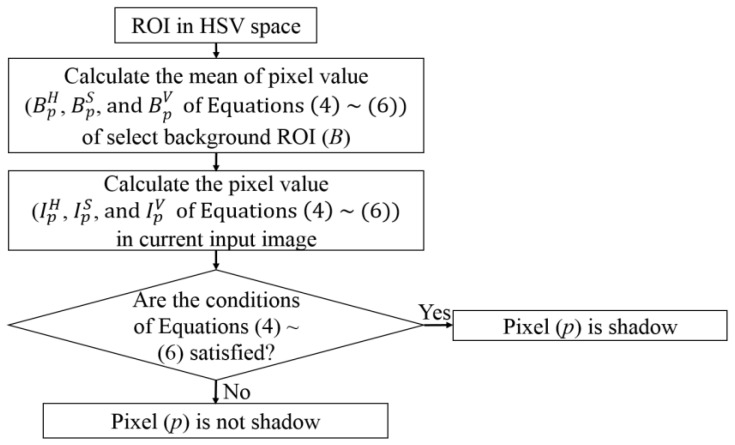
Flowchart for determining shadow for feature 1.

**Figure 6:**
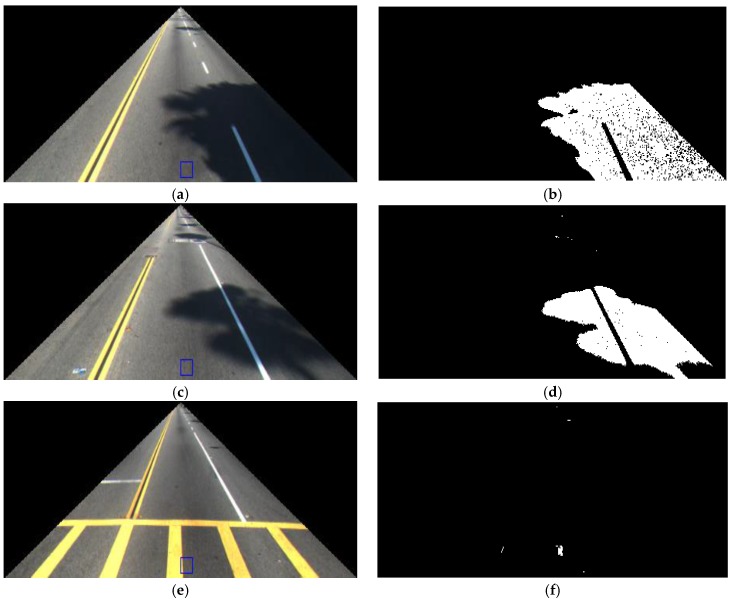
Examples of extracted shadows for calculating feature 1. Background ROI (*B*) of Equations (4)–(6) and Figure 5 is shown by the blue box in Figure 6a,c,e: (**a**,**c**,**e**) Image in the ROI; (**b**,**d**,**f**) binarization image of detected shadow by Figure 5.

Figure 5 shows the flowchart for determining shadow for feature 1. As the first step of Figure 5, the ROI of RGB color space is converted to that of HSV color space [35]. In the HSV color space, the *V* component is a direct measure of intensity. Pixels that belong to shadow should have a lower value of *V* than those in the nonshadow regions, and the hue (H) component of shadow pixels changes within a certain limited range. Moreover, shadow usually lowers the saturation (S) component. In conclusion, a pixel *p* is considered to be part of shadow if its value is satisfactory with the following three equations [36]:

(4) $$thr_{Valpha}\leq \frac{I_{p}^{V}}{B_{p}^{V}}\leq thr_{Vbeta}$$

(5) $$I_{p}^{S}-B_{p}^{S}\leq thr_{S}$$

(6) $$\left|I_{p}^{H}-B_{p}^{H}\right|\leq thr_{H}$$

where $I_{p}^{E}$ and $B_{p}^{E}$ represent the specific channel of HSV color space (*E* = *H*, *S*, and *V*, respectively) for the pixel p in the current input image (*I*) and in the background ROI (*B*) (blue boxes of Figure 6a,c,e), respectively. The values $thr_{Valpha},\;thr_{Vbeta},\;thr_{S},\;\mathrm{and}\;thr_{H}$ represent the threshold values, and these values are respectively 0.16, 0.64, 100, and 100. These optimal values were empirically determined by experiments with training data. It is unnecessary to recalculate the thresholds even if the camera is modified. In our experiment of Section 4, we used same thresholds with three different databases where the different cameras were used. Among these thresholds, those which affect shadow detection most are $thr_{Valpha}$ and $thr_{Vbeta}$, because $thr_{Valpha}$ is used to define a maximum threshold for the darkening effect of shadows on the background pixel, whereas $thr_{Vbeta}$ prevents the system from incorrectly identifying the too dark (nonshadow) pixels as shadow pixels [37].

From the ROI of Figure 3, the ROI for lane detection is reduced by removing the left and right upper areas of the images as shown in Figure 6a,c,e to extract the features used as the input to FIS. Figure 6b,d,f shows the binarization image of extracted shadow within these ROIs based on Equations (4)–(6) and Figure 5. Thus, the average number of shadow pixels in this ROI is calculated as feature 1 in our research.

### 3.4. Calculating Feature 2 (Gray Difference Based on Global Background Area)

**Figure 7:**
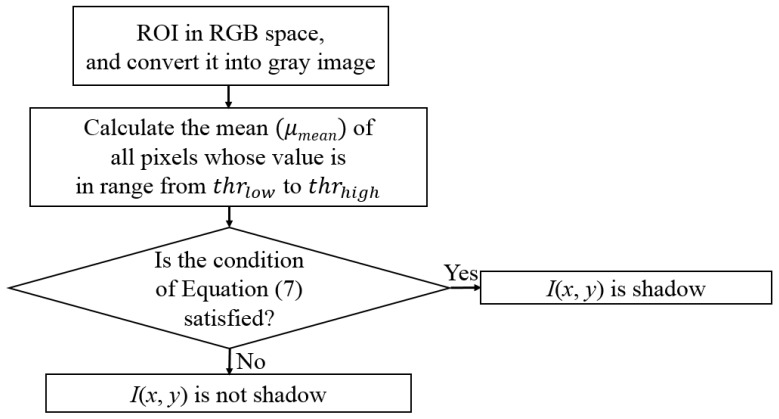
Flowchart for determining shadow for feature 2.

**Figure 8:**
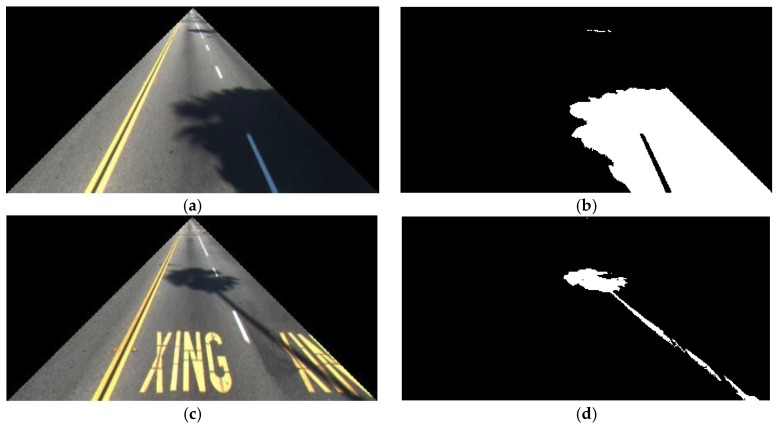
Examples of extracted shadows for calculating feature 2: (**a**,**c**) Image in the ROI; (**b**,**d**) binarization image of detected shadow by Figure 7.

Figure 7 shows the flowchart for determining shadow for feature 2. While feature 1 is calculated in HSV color space, feature 2 is calculated in gray image to consider the characteristics of shadow in various color and gray spaces. Two thresholds for lower and upper bound threshold $thr_{low}$ and $thr_{high}$ are determined to calculate feature 2. According to the kinds of experimental databases, the threshold values are a little changed, and the ranges of these two thresholds are 16~17 and 48~50, respectively. These ranges of optimal thresholds were empirically determined by experiments with training data. Next, the mean value of all pixels whose value is in the range from $thr_{low}$ to $thr_{high}$ is calculated as $\mu_{mean}$. For example, there are four pixels inside the ROI of Figure 8, and their pixel values (gray levels) are 20, 15, 33, and 40, respectively. Because three pixels of 20, 33, and 40 (except for 15) belong to the range from $thr_{low}$ to $thr_{high}$, $\mu_{mean}$ is calculated as 31((20 + 33 + 40)/3). Finally, the pixel (*x*, *y*) which satisfied the condition of Equation (7) is determined as shadow:

(7) $$\left|I\left(x,y\right)-\;\mu_{mean}\right|\leq thr_{medium}$$

where I(x,y) is the pixel value at coordinate *x* and *y* in the ROI for lane detection of Figure 8a,c; and the optimal threshold ($thr_{medium}$) was also empirically determined by experiments with training data. According to the kinds of experimental databases, the threshold value is a little changed, and the range of this threshold is 24~26, respectively. Next, the average number of shadow pixels in this ROI is calculated as feature 2 in our research.

According to the position of camera, the detected position of vanishing point can be changed in the input image and the consequent ROI of Figure 8 can be also changed, which can influence the threshold values of Figure 7. However, the changes of threshold values are not large as explained above, and for the experiments of Section 4, we used the similar threshold values in three different databases of the Caltech dataset, Santiago Lanes Dataset (SLD), and Road Marking dataset where the positions of cameras are different.

### 3.5. Designing Fuzzy Membership Functions and Rule Table

For the next step, our method measures the level of shadow included in the ROI by using FIS using two features (features 1 and 2) as inputs as shown in Figure 1. The range of each feature is represented from 0 to 1 by min-max scaling to use two features as inputs to FIS. The input values are separated into two classes (low (L) and high (H)) in the membership function. In general, there is an overlapped area between these two value classes, and we define the shape of the input membership function as a linear function. Linear membership functions have been widely adopted in the FIS because the algorithm is less complex and the calculation speed is very fast compared to the nonlinear membership function [38,39,40]. With the training data, we obtained the distributions of features 1 and 2, and based on maximum entropy criterion, we designed the input member ship functions as follows:

(8) $$F_{L\_feature\;i}\left(x\right)=\{\begin{array}{c} \begin{array}{c} \begin{array}{cc} 1 & \;\mathrm{for}\;0\leq x\leq p_{L\_i} \end{array}\\ a_{L\_i}x+b_{L\_i}\;\mathrm{for}\;p_{L\_i}\leq x\leq q_{L\_i} \end{array}\\ \begin{array}{cc} 0 & \;\mathrm{for}\;q_{L\_i}\leq x\leq 1 \end{array} \end{array}$$

(9) $$F_{H\_feature\;i}\left(x\right)=\{\begin{array}{c} \begin{array}{c} \begin{array}{cc} 0 & \;\mathrm{for}\;0\leq x\leq p_{H\_i} \end{array}\\ a_{H\_i}x+b_{H\_i}\;\mathrm{for}\;p_{H\_i}\leq x\leq q_{H\_i} \end{array}\\ \begin{array}{cc} 1 & \;\mathrm{for}\;q_{H\_i}\leq x\leq 1 \end{array} \end{array}$$

where $a_{L\_i}$ is 1/($p_{L\_i}-q_{L\_i}$) and $b_{L\_i}\;$ is $q_{L\_i}$/($q_{L\_i}-p_{L\_i}$). In addition, $a_{H\_i}$ is 1/($p_{H\_i}-q_{H\_i}$) and $b_{H\_i}$ is $q_{H\_i}$/($q_{H\_i}-p_{H\_i}$). In Equations (8) and (9), *i* = 1 and 2, and $F_{L\_feature\;i}\left(x\right)$ is the L membership function of feature *i*, whereas $F_{H\_feature\;i}\left(x\right)$ is its H membership function. Next, we can obtain the following equations:

(10) $$Prob_{L\_feature\;i}=\sum_{x=0}^{1}F_{L\_feature\;i}\left(x\right)Dist_{L\_feature\;i}(x)$$

(11) $$Prob_{H\_feature\;i}=\sum_{x=0}^{1}F_{H\_feature\;i}\left(x\right)Dist_{H\_feature\;i}(x)$$

**Figure 9:**
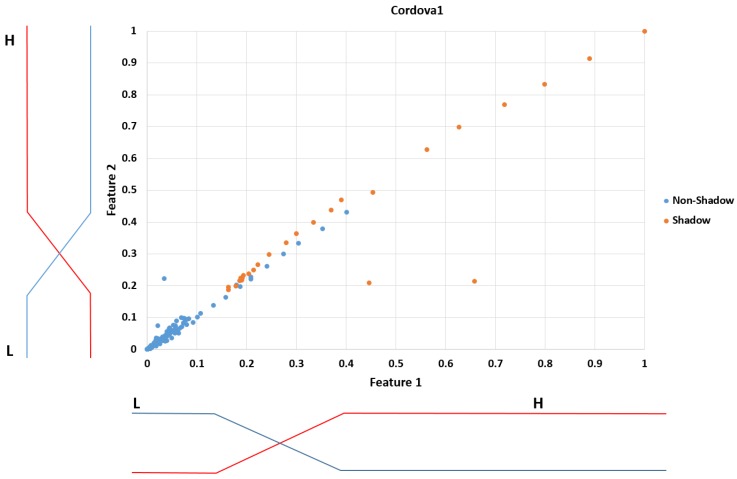
Input fuzzy membership functions for features 1 and 2, which are designed by maximum entropy criterion with training data.

In Equations (10) and (11), *i* = 1 and 2. In addition, $Dist_{L\_feature\;i}\left(x\right)$ is the L (data) distribution of feature *i* (nonshadow data of Figure 9), whereas $Dist_{H\_feature\;i}\left(x\right)$ is the H (data) distribution of feature *i* (shadow data of Figure 9). Based on Equations (10) and (11), the entropy can be calculated as follows:

(12) $$H\left(p_{L_{i}},\;q_{L_{i}},\;p_{H_{i}},\;q_{H_{i}}\right)=-Prob_{L\_feature\;i}\log\left(Prob_{L\_feature\;i}\right)-\;Prob_{H\_feature\;i}\log\left(Prob_{H\_feature\;i}\right)$$

where *i* = 1 and 2. Based on the maximum entropy criterion [41,42], the optimal parameters of $\left(p_{L\_i},\;q_{L\_i},\;p_{H\_i},\;q_{H\_i}\right)$ of feature *i* are calculated by being selected when the entropy $H\left(\;p_{L\_i},\;q_{L\_i},\;p_{H\_i},\;q_{H\_i}\right)$ is maximized. From this, the input membership functions of features 1 and 2 are defined as shown in Figure 9.

**Figure 10:**
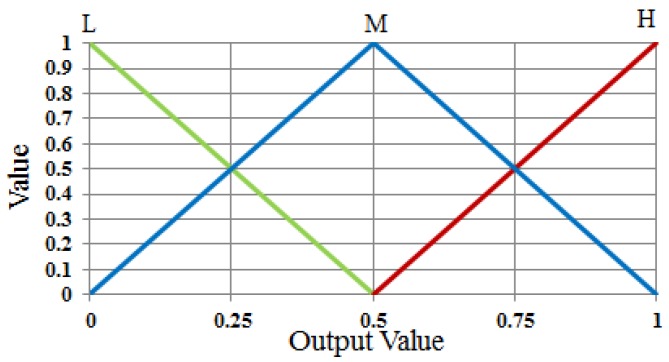
Output membership functions.

These membership functions are used to convert input values to a degree of membership. The output value of FIS is also described in the form of a linear function from the membership function to determine whether selected ROI contains more shadow or less. In our research, we designed the output membership function using three functions of low (L), medium (M), and high (H) as shown in Figure 10. We define the output fuzzy rule as “L” in the case when the level of shadow is close to 0 (minimum) and “H” when the level of shadow is close to 1 (maximum), as shown in Table 2. Thus, the optimal output value of FIS can be obtained using these output membership functions: the fuzzy rule table, and the combination of the defuzzification method with Min and Max rules.

### 3.6. Determining Shadow Score Based on Defuzzification Methods

**Figure 11:**
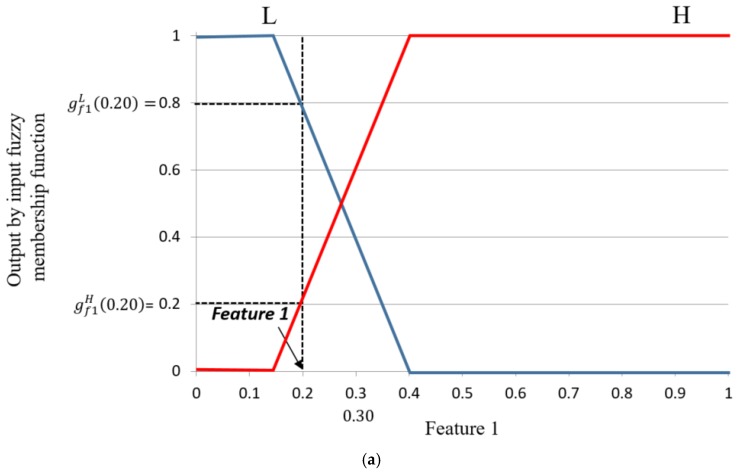

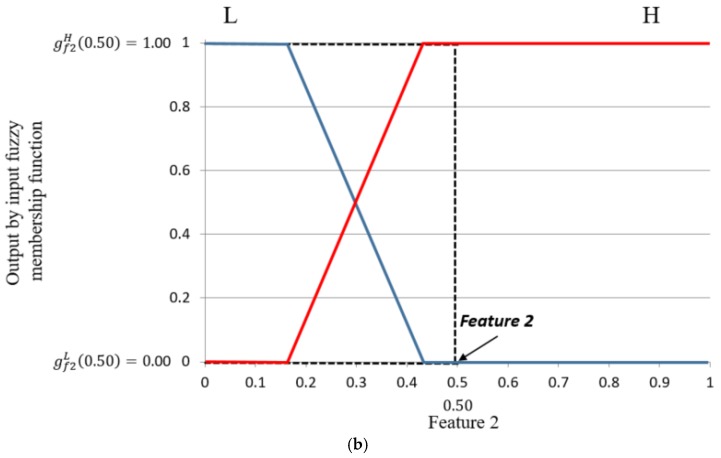
Obtaining the output value of the input membership function for two features: (**a**) feature 1; (**b**) feature 2.

Using the two normalized input features, four corresponding values can be calculated using the input membership functions as shown in Figure 11. Four functions are defined as $g_{f1}^{L}\left(\cdot \right),\;g_{f1}^{H}\left(\cdot \right),\;g_{f2}^{L}\left(\cdot \right),$ and $g_{f2}^{H}\left(\cdot \right)$. The corresponding output values of the four functions with input values of *f*1 (feature 1) and *f*2 (feature 2) are shown by $\;(g_{f1}^{L},\;g_{f1}^{H})\;\mathrm{and}\;(g_{f2}^{L},$ $g_{f2}^{H})$. For example, suppose that the two input values for *f*1 and *f*2 are 0.20 and 0.50, respectively, as shown in Figure 11. The values of $(g_{f1}^{L},\;g_{f1}^{H})\;\mathrm{and}\;(g_{f2}^{L},$ $g_{f2}^{H})$ are (0.80(L), 0.20(H)) and (0.00(L), 1.00(H)), respectively, as shown in Figure 11. With these values, we can obtain the following four combinations: (0.80(L), 0.00(L)); (0.80(L), 1.00(H)); (0.20(H), 0.00(L)); and (0.20(H), 1.00(H)).

With these four combinations, a value is selected by the Min or Max rule with the fuzzy rules in Table 2. In the Min method, the minimum value is selected from each combination, whereas the Max method selects the maximum value. For example, for (0.80(L), 1.00(H)), in the case of the Min rule, 0.80 is selected and M is determined (if “L” and “H,” then “M” as shown in Table 2). Finally, the obtained value is 0.80(M). In the case of the Max rule, 1.00 is selected with M, and the obtained value is 1.00(M). These obtained values are called “inference values” (IVs). Table 3 shows the obtained IVs by the Min or Max rule with the rule table of Table 2 from these four combinations of (0.80(L), 0.00(L)); (0.80(L), 1.00(H); (0.20(H), 0.00(L)); and (0.20(H), 1.00(H)).

**Figure 12:**
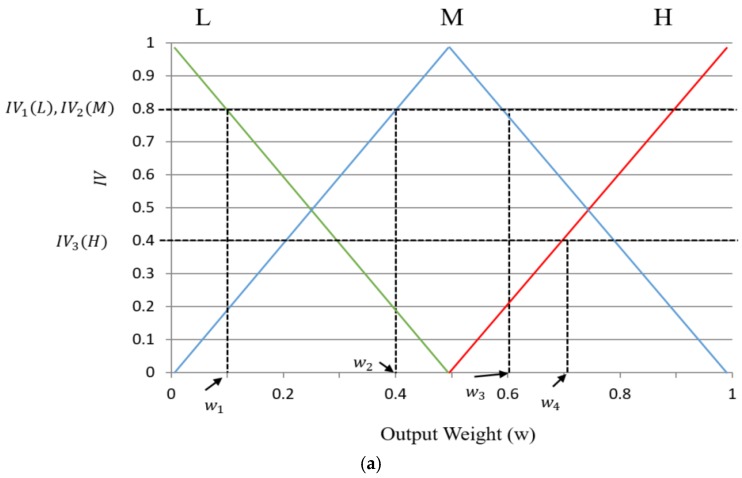

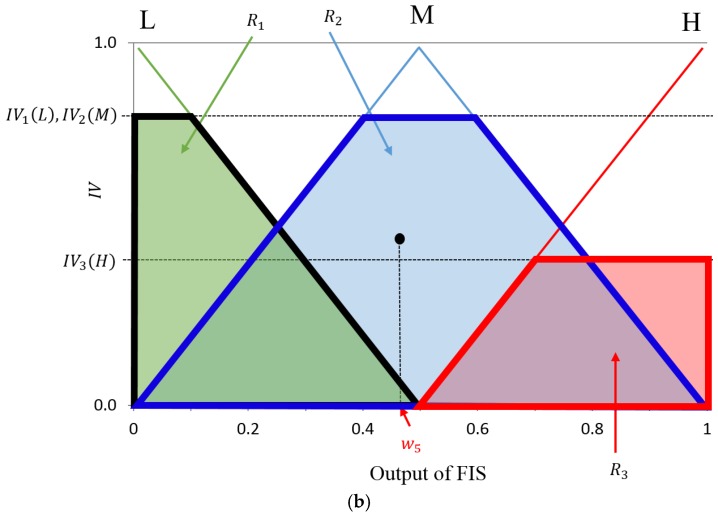
Output score values of FIS using defuzzification methods. (**a**) FOM, LOM, and MOM; (**b**) COG.

Using four IVs, we can obtain the final output of FIS by one of the five defuzzification methods. In our research, we only consider five methods for defuzzification: first of maxima (FOM), last of maxima (LOM), middle of maxima (MOM), mean of maxima (MeOM), and center of gravity (COG) [38,43,44]. The FOM method selects the minimum value ($w_{1}$) among the values calculated using the maximum IV (($IV_{1}\left(L\right)$ and $V_{2}\left(M\right)$ of Figure 12a), LOM selects the maximum value ($w_{3})$ among the values calculated using the maximum IV (($IV_{1}\left(L\right)$ and $IV_{2}\left(M\right)$). The MOM gets the middle value of the weight value from FOM and LOM ($\left(w_{1}+w_{3}\right)/2)$, and MeOM gets the mean value ($\left(w_{1}+w_{2}+w_{3}\right)/3$). The output of FIS obtained by the COG is $w_{5}$ as represented in Figure 12b, which is calculated from the COG of three regions ($R_{1},\;R_{2},\;\mathrm{and}\;R_{3})$. We compared the five defuzzification methods and used one method (COG) which shows the best performance. That is, $w_{5}$ is used as $fuzzy_{score}$ of the Equations (13) and (14) to adaptive change the parameters of LSD and CannyLines detector.

### 3.7. Adaptively Change Input Parameters for Line Segment Detector Algorithms

The obtained output of FIS in Section 3.6 represents the level of shadow in the input image, and then based on this output, the input parameters of line segment detector algorithms are changed adaptively, as shown in Equations (13) and (14). That is because more line segments are usually extracted from the boundaries of shadows in the case when the image including the larger level of shadows is compared to the image including the lesser level of shadows.

In this paper, we combine two robust line segment detection algorithms to efficiently detect road lane markings boundaries from an input image. They are called LSD algorithm [32,33] in OpenCV library [45] and CannyLines detector [34], which are applied into the ROI of the input image, sequentially. The LSD method has several parameters to control meaningful line segments as follows; and the scale is adjusted in our research because it affects line segment detection more than sigma_scale:

(1) Scale (α of Equation (13)): The scale of the image that is used to find the lines; its range is from 0 to 1. The 1 means that the original image is used for line segment detection. A smaller value shows that the image of a smaller size is used for line segment detection. For example, 0.5 means the image whose width and height are respectively half compared to those of the original image is used for line segment detection
(2) Sigma_scale: Sigma value for Gaussian filter

Based on the output of FIS, we update the LSD parameter (scale) dynamically based on Equation (13). In this Equation, $\alpha_{0}$ is the default scale (0.8) of the LSD parameter, and $fuzzy_{score}$ is the output of FIS, whose range is from 0 to 1. The image of larger $fuzzy_{score}$ means that the larger levels of shadows are included. Therefore, in this case, we use the smaller α for LSD, which means the image size is reduced for line segment detection. With the image of smaller size, the high frequency edges of the image disappear compared to that of larger size. Therefore, the line segments from the boundary of the shadow tends to be reduced:

(13) $$\alpha =(\alpha_{0}+0.2)-fuzzy_{score}$$

Most of the parameters of the CannyLines detector related to the input image are determined by the image itself. However, there are still some parameters which can be adjusted, and $\mu_{v}$ is adjusted in our research because it affects line segment detection more than other parameters:

(1) $\mu_{v}$: Denotes the lower limit of a gradient magnitude
(2) $\theta_{s}$: Represents the minimal length of an edge segment to be considered for splitting and equals twice that of the possibly shortest edge segment
(3) $\theta_{m}$: Represents the maximal direction deviation tolerance of two close-direction line segments to be considered for merging

Based on the output of FIS, the parameter of the CannyLines detector is also updated by Equation (14). The value $\mu_{0}$ is the default value (70) of the lower limit of a gradient magnitude. As explained previously, the image of larger $fuzzy_{score}$ means that the larger levels of shadows are included. Based on Equation (14), consequently, $\mu_{v}$ becomes larger. A larger $\mu_{v}$ means that the higher limit of a gradient magnitude is used, which causes the reduction of the detected line segment by the CannyLines detector:

(14) $$\mu_{v}=\mu_{0}\cdot 10\cdot fuzzy_{score}$$

**Figure 13:**
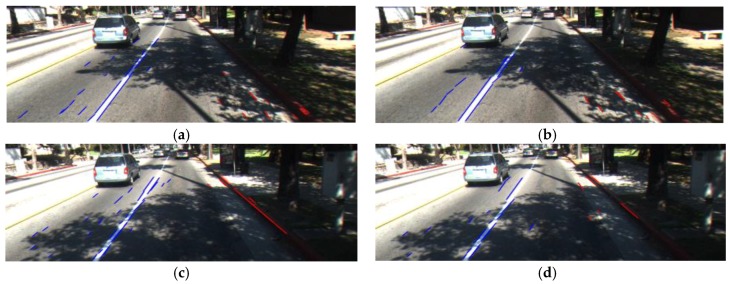
Examples of comparison between before and after adaptively adjusting the parameters by the output of FIS: (**a**) Default parameters for LSD; (**b**) Adjusted parameters for LSD; (**c**) Default parameters for CannyLines detector; (**d**) Adjusted parameters for CannyLines detector.

As shown in Figure 13, through the adaptive adjusting of parameters of the LSD and CannyLines detector, we can find that the incorrect line segments are reduced in the result image.

### 3.8. Detecting Correct Lane Boundaries by Eliminating Invalid Line Segments Based on Angle and Vanishing Point

As shown in Figure 13b,d, there are still incorrect line segments after adaptively adjusting the parameters by the output of FIS. Therefore, in the next step, incorrect line segments are removed based on the characteristics of the road lane.

**Figure 14:**
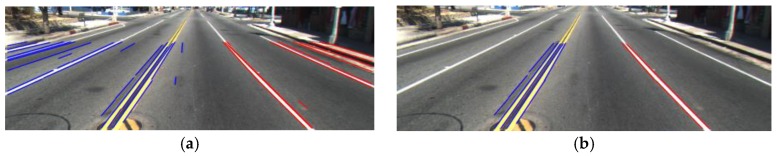
Number of detected line segment based on the angle condition: (**a**) Before using the angle condition; (**b**) After using the angle condition.

Because the car always operates between two road lanes, left and right road lane markings appear like two sides of a trapezoid in the image as shown in Figure 13. Therefore, only the left and right road lanes that satisfy the angle condition are maintained, regardless of their location [6]. In detail, we separate the ROI into two areas of left and right-side ROIs based on the middle position in the horizontal direction of ROI. That is, we decide that all line segments whose starting point has an *x*-coordinate of the range [0, $\frac{W_{ROI}}{2}-1]$ belong to the left side-ROI; whereas, all the others belong to the right-side ROI. Here, $W_{ROI}$ is the width of the ROI. Then, we define empirically the range of angle of the road lane for left side-ROI and right-side ROI as $\theta_{left}\left(25^{{}^{\circ}}-75^{{}^{\circ}}\right)$ and $\theta_{right}\left(105^{{}^{\circ}}-155^{{}^{\circ}}\right),$ respectively. Any line segments whose angle does not belong to these ranges ($\theta_{left}\left(25^{{}^{\circ}}-75^{{}^{\circ}}\right)$ and $\theta_{right}\left(105^{{}^{\circ}}-155^{{}^{\circ}}\right)$) are removed. As shown in Figure 14, incorrect line segments are removed after using the angle condition.

**Figure 15:**
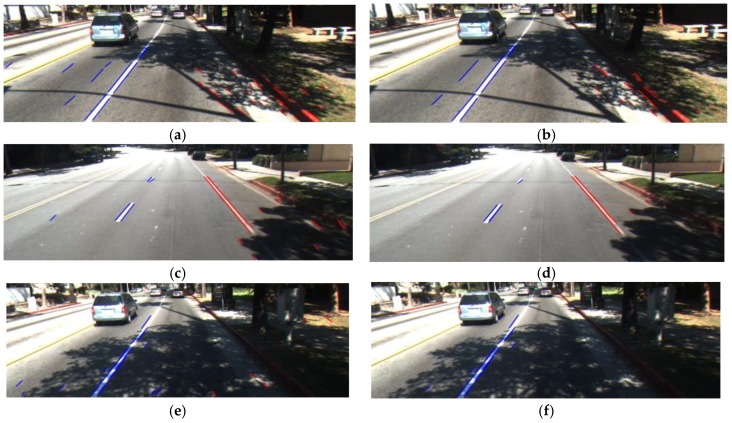
Remove irrelevant line segments based on the vanishing point condition: (**a**,**c**,**e**) Before using the vanishing point condition; (**b**,**d**,**f**) After using the vanishing point condition.

There are still incorrect line segments after using the angle condition as shown in Figure 15a,c,e. Therefore, we use the vanishing point condition to remove these line segments. As explained in Section 3.2, all left and right boundaries of road lane markings intersect at a point called the vanishing point. Once the vanishing point is detected, we can obtain its *x* and *y* coordinates as $x_{vp}$ and $y_{vp}$. Next, we can calculate slope a and *y*-intercept b of each detected line segment, and calculate the linear equation of this straight line with $x_{vp}$ to get the *y* coordinate value. Finally, we compare the distance value between $y_{vp}$ and the *y* coordinate value by using the linear equation of a straight line with a certain threshold value as shown in Equation (15), and remove the line segments if this distance value exceeds a certain threshold value. Figure 15b,d,f shows the results by using the vanishing point condition:

(15) $$\left|y_{vp}-\left(a\cdot x_{vp}+b\right)\right|\leq thr_{dist}$$

**Figure 16:**
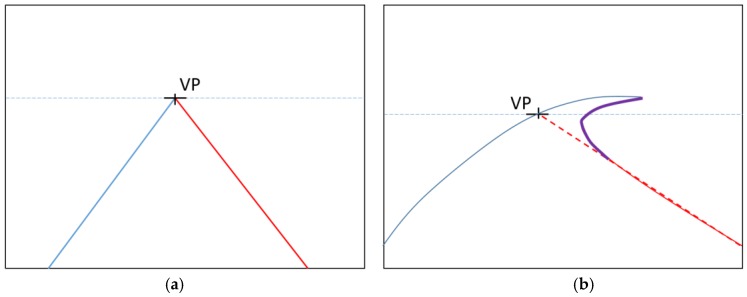
Detected vanishing point. VP means the detected vanishing point: (**a**) straight road lane markings; (**b**) curved lane markings.

In the case of a curved lane, the angle condition is not valid. For example, in Figure 16b the angle of the right lane of the upper region is similar to that of the left lane by the curved road. Therefore, the above angle condition is applied only in the middle and lower areas of ROI. In the upper area of ROI, the line segment whose angle is much different from that of the line segment detected below the region is removed. Detailed algorithms are referenced in [6].

However, in our research, the curved lanes are not detected correctly because of the vanishing point. This problem is depicted in Figure 16b. Based on the vanishing point condition, we only keep line segments that have an extension crossing the vanishing point; thus, we cannot detect the whole curved lane marking, but the part of the curved lane (of Figure 16b) can be removed by the vanishing point condition. To solve this problem, we apply the vanishing point condition only in the lower areas (below the violet line of Figure 16b) of ROI based on the detected vanishing point.

After eliminating the line segments according to angle and vanishing point conditions, multiple groups of line segments that belong to road lane markings remain. In this final step, we use methods similar to those that were used in [6] to combine small fragmented line segments into a single line. We define a $3^{{}^{\circ}}$ of angle difference and three of the Euclidean distance difference as the stopping conditions, which means that we concatenate any two adjacent lines that have smaller than $3^{{}^{\circ}}$ and three pixels of angle difference and the Euclidean distance difference, respectively.

## 4. Experimental Results

**Figure 17:**
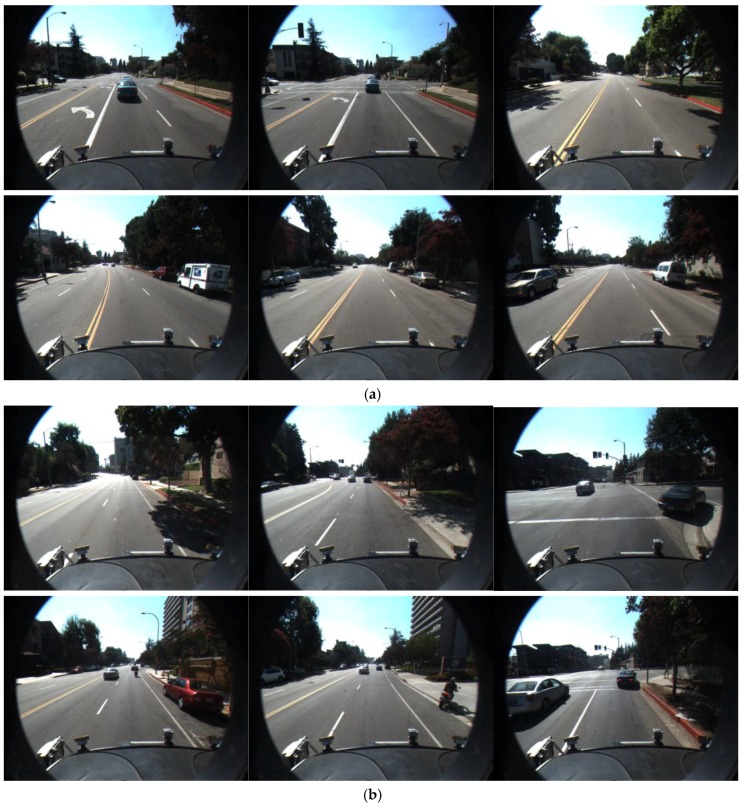

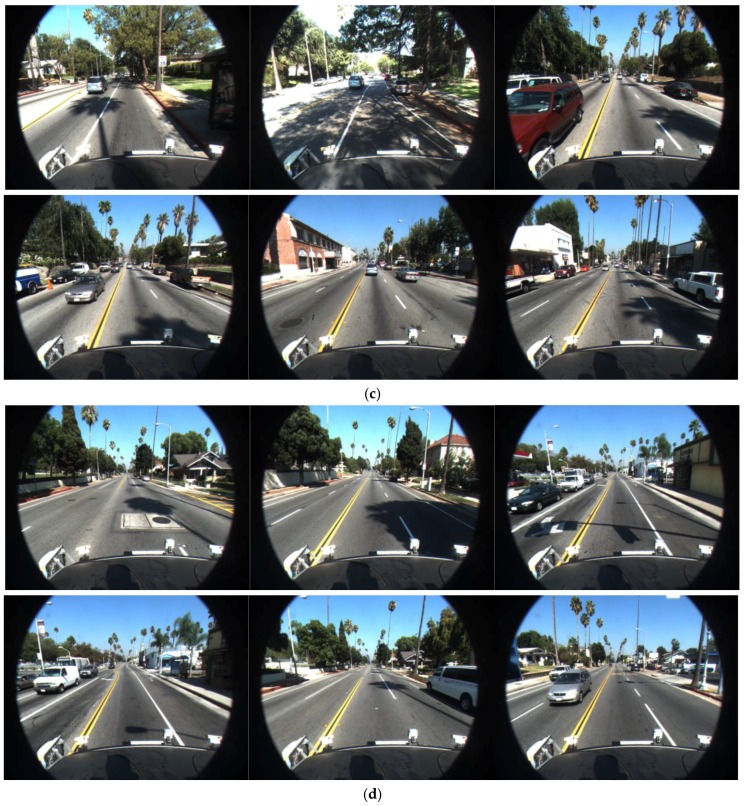
Examples of the Caltech dataset: (**a**) Cordova 1; (**b**) Cordova 2; (**c**) Washington 1; and (**d**) Washington 2.

**Figure 18:**
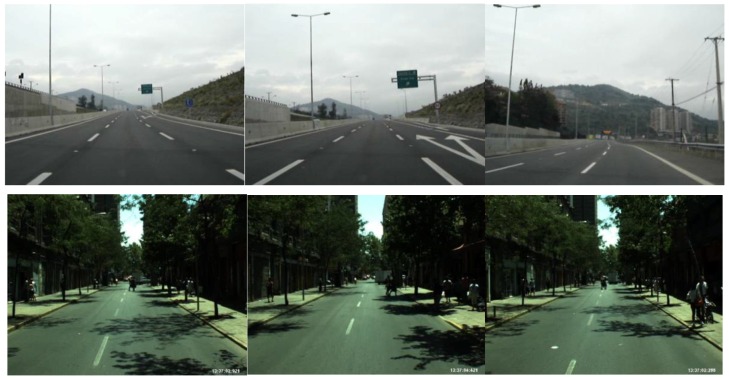
Examples of the SLD dataset.

**Figure 19:**
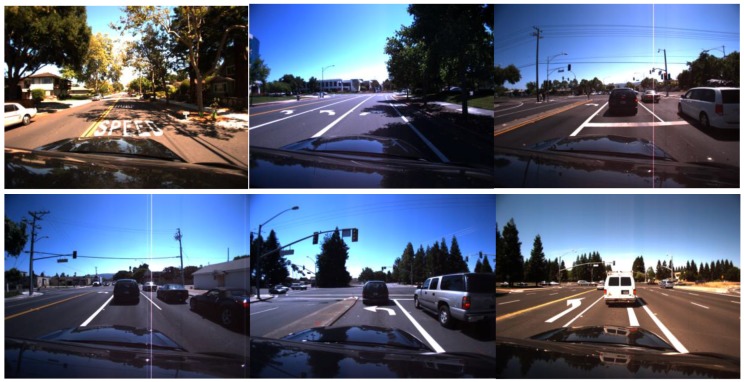
Examples of the Road Marking dataset.

We tested our proposed method with various datasets as shown in Figure 17, Figure 18 and Figure 19. For the Caltech dataset, 1016 images were used, and the size of the image was 640 × 480 pixels [5]. For the Santiago Lanes Dataset (SLD), 1201 images with the size of 640 × 480 pixels were used [46]. In addition, the Road Marking dataset consists of various subsidiary dataset with more than 3000 frames captured under various illumination conditions, and the image size is 800 × 600 pixels [47,48]. These databases were collected at different times along the day. We performed the experiments on a desktop computer with Intel Core^TM^ i7 3.47 GHz, 12 GB memory of RAM, and the algorithm was implemented by Visual C++ 2015 and OpenCV library (version 3.1).

The ground-truth (starting and ending) positions of road lane markings were manually marked in the images to measure the accuracy of lane detection. Because our goal is to discriminate dashed and solid lanes in addition to lane detection, we manually detect the ground-truth point, and then compare it with detected starting and ending points with a certain interdistance threshold value to determine whether the detected line is correct or not.

In our method, we only consider whether the detected line segment is a lane mark or not, so negative data do not occur (i.e., ground-truth data of a non-lane), and true negative (TN) errors are 0% in our experiments. Other kinds of errors such as true positive (TP), false positive (FP), and false negative (FN) are defined and calculated to obtain precision, recall, and F-measure as shown in Equations (16)–(18) [49,50]. The number of TP, FP, and FN are represented as #TP, #FP and #FN, respectively:

(16) $$\mathrm{Precision}=\frac{\#\mathrm{TP}}{\#\mathrm{TP}+\#\mathrm{FP}}$$

(17) $$\mathrm{Recall}=\frac{\#\mathrm{TP}}{\#\mathrm{TP}+\#\mathrm{FN}}$$

(18) $$F-\mathrm{measure}=2\times \frac{\mathrm{Precision}\cdot \mathrm{Recall}}{\mathrm{Precision}+\mathrm{Recall}}$$

Table 4, Table 5 and Table 6 show the accuracies of our method with each dataset.

**Figure 20:**
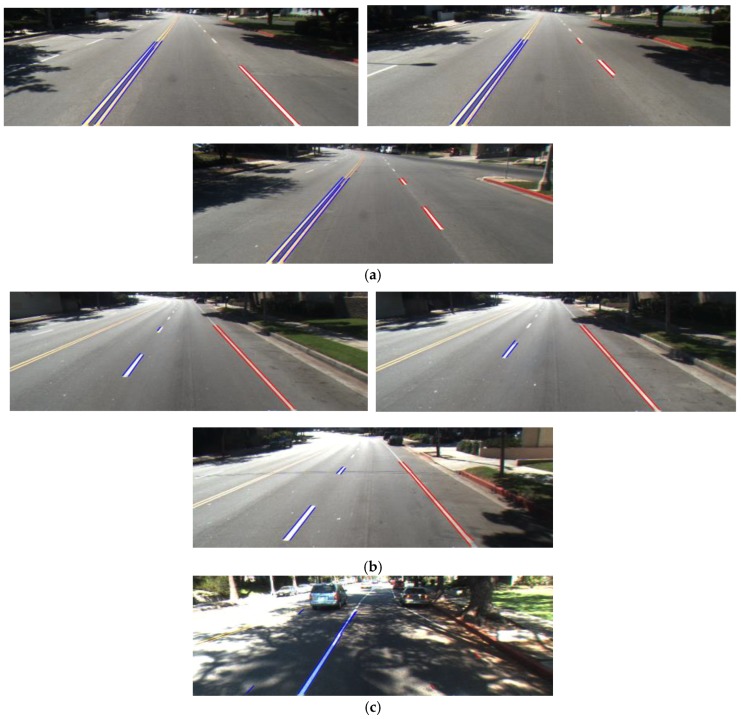

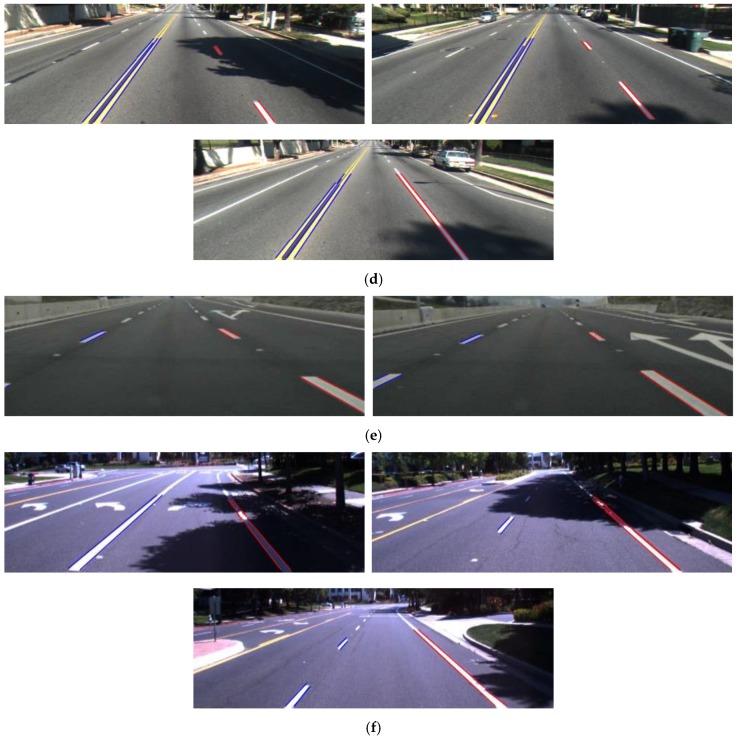
Correct lane detection: (**a**–**d**) Caltech dataset ((**a**) Cordova 1; (**b**) Cordova 2; (**c**) Washington 1; (**d**) Washington 2); (**e**) SLD dataset; (**f**) Road marking dataset.

**Figure 21:**
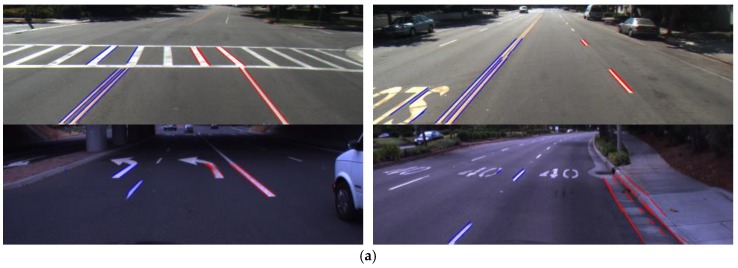

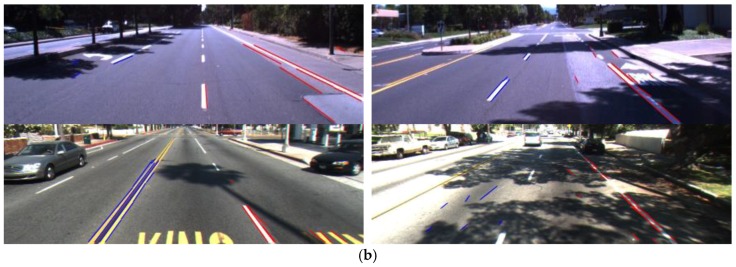
Incorrect lane detection due to (**a**) nonroad lane objects, and (**b**) shadow.

Figure 20 shows correct lane detection using our method with various datasets. In addition, Figure 21 shows some examples of incorrect detection results. In Figure 21a, our method incorrectly recognized non-road lane objects such as crosswalks, road-signs, text symbols, and pavement as lane markings. In those cases, there are no dynamic conditions to distinguish which one belongs to a road lane and which one belongs to non-road lane objects. In addition, Figure 21b shows the effect of shadows on our method. Although our method uses the fuzzy rule to determine the amount of shadow in the image to automatically change the lane detector parameter, it still fails in some cases where extreme illumination occurs.

**Figure 22:**
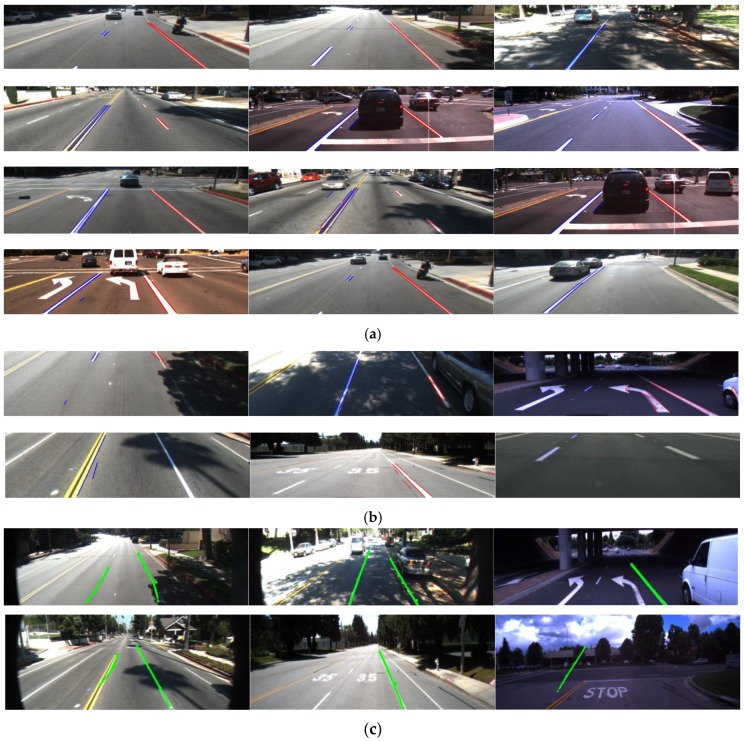

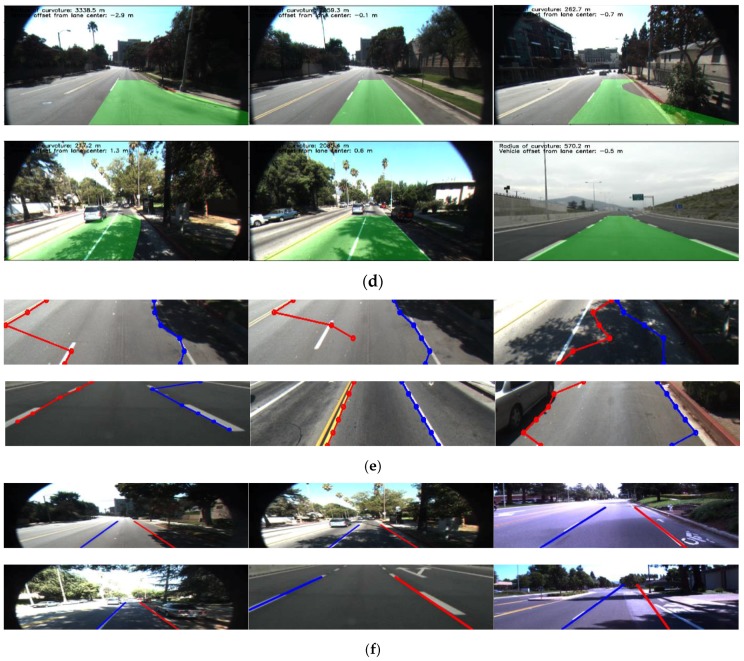
Comparison of lane detection: (**a**) our method; (**b**) Hoang et al.’s method [6]; (**c**) Aly method [5]; (**d**) Kylesf method [7]; (**e**) Truong et al.’s method [4]; (**f**) Nan et al.’s method [1].

In the next experiment, we compare the performance of our method with some other methods: the Hoang et al. method [6], Aly method [5], Truong method [4], Kylesf method [7] and Nan method [1]. In [6], the line segment was detected by the LSD algorithm to detect the road lane. However, in [6], the lane detection was performed within the smaller ROI compared to the ROI in our research, and the number of images including shadows is smaller than that in our research. Therefore, the accuracies of lane detection, even with the same database using the methods [6] in Table 7, are lower than those reported in [6]. Owing to the same reasons, the accuracies by the methods [4,5] reported in [6] are different from those in Table 7. In other methods, they converted the input image by IPM with HT [5,7] to detect a straight line, and the random sample consensus (RANSAC) algorithm [5] to fit lane makers. We empirically found the optimal thresholds for these methods [1,4,5,6,7]. As shown in Table 7 and Figure 22, our method outperforms previous methods. The reason why the accuracies by [1,4,5,7] are too low is that they did not detect the left and right boundaries of road lane, and did not discriminate the dashed and solid lanes. That is, their method did not detect the starting point and ending point of road marking as well as the left and right boundaries of road lane. Although the method [6] has these two functionalities, their method is more affected by the shadows in the image, and the accuracies by [6] are lower than ours. Moreover, this method [6] uses fixed ROI for detecting road lane and does not detect the vanishing point; thus, it generates more irrelevant line segments. That is why precision by this method is lower than that by our method. As shown in Figure 22a, we included the examples with the presence of vehicles on the same road lane of the detection vehicle. These cases were already included in our experimental databases. As shown in Figure 22a and Table 7, the presence of cars on the same road lane does not affect our detection results.

As the next experiment, we measured the processing time per frame by our method as shown in Table 8. As shown in Table 8, we can confirm that our method can be operated at a fast speed (about 40.4 frames/s (1000/24.77)).

In other previous researches [51,52,53,54], they showed the high performance of road lane detection irrespective of various weather conditions, traffic, and curved lanes, etc. However, they did not discriminate the solid and dashed lanes in the detected road lanes although it is necessary for autonomous vehicle. Different from them, even the solid and dashed lanes are discriminated in the detected road lanes by our method. In addition, more severe shadows are considered in our research compared to the examples of three results in [51,52,53,54]. In other methods [55,56], they can detect the road lane in difficult environments, but the method [55] did not discriminate the solid and dashed lanes in the detected road lanes either. The method [56] discriminated the solid and dashed lanes in the detected road lanes. However, they did not detect the exact starting and ending positions of all the dashed lanes although the accurate detection of these positions are necessary for the prompt or predictive decision of the moment of crossing road lane by fast moving autonomous vehicle. Different from them, in addition to the discrimination of the solid and dashed lanes, the accurate starting and ending positions of dashed lane are also detected by our method.

## 5. Conclusions

In this study, we proposed a method to overcome severe shadows in the image, for obtaining better road lane detection results. We used two features as the inputs for FIS: HSV color difference based on local background area (feature 1) and gray difference based on global background area (feature 2) for evaluating the level of shadow in the ROI of a road image. Two features from different color and gray spaces were used for FIS for considering the characteristics of shadow in various color and gray spaces. Using FIS based on these two features, we estimated the level of shadows based on the output of FIS after the defuzzification process. We modeled the input membership functions based on the training data of two features and maximum entropy criterion for enhancing the accuracy of FIS. By adaptively changing the parameters of LSD and CannyLines detector algorithms based on the output of FIS, more accurate line detection was possible based on the fusion of the detection results by LSD and CannyLines detector algorithms, irrespective of severe shadows on the road image. Experiments with three open databases showed that our method outperformed previous methods, irrespective of severe shadows in the images. Because tracking information in successive image frames was not used in our method, the detection of lanes by our method was not affected by the speed of the car.

However, complex traffic with the presence of cars can affect our performance when detecting vanishing points and line segments, determining shadow levels, and locating final road lanes, which is the limitation of our system. Nevertheless, our three experimental databases do not include these cases, and we could not measure the effect of the presence of cars on the performance of our system.

In future, we would collect our own database including the complex traffic with the presence of cars, and measure the effect of these cases on our performance. In addition, we plan to solve this limitation by deep learning-based lane detection. Also, we plan to use a deep neural network for discriminating dashed and solid lane markings under various illumination conditions, as well as for detecting both straight and curved lanes. In addition, we would research to combine our method with a model-based method to enhance the performance of lane detection.

## Figures and Tables

**Table 1:** Comparisons of previous and proposed methods on road lane detection.

Category	Model-Based Methods	Feature-Based Methods	
		Not Considering Severe Shadows on Road Image	Considering Severe Shadows on Road Images (Proposed Method)
**Methods**	- B-spline model [4,8,9,10,11] - Parabolic model [12] - Local road model or geometrical model [13] - Quadratic curve model [14,19] - IPM [5,15,16,17,18,29]	- Using edge features [30], EDLines method [31], and illumination invariant lane features [27] - SCA, fuzzy C-mean and fuzzy rules in YCbCr space [25] - Canny edge detector and HT [18] - Fuzzy C-mean in HSI color space [28] - Line segment detector [6] - Convolutional neural network (CNN) [22]	FIS-based estimation of the level of shadows and adaptive change of the parameters of LSD and CannyLines detector algorithms
**Advantages**	High performance and accuracy of road lane detection by using mathematical models	- Performance is not affected by the model parameters or the initial parameters of the camera - Algorithm is simple with fast processing speed	Accurate road lane detection can be possible irrespective of severe shadows on road image
**Disadvantages**	It works well only when complete initial parameters of the camera or the structure of the road are provided	It works well only in visible and clear road conditions where the road lane markings can be easily separated from the ground by enhancing the contrast and brightness of the image	Additional procedure for designing fuzzy membership function and fuzzy rule tables is necessary

**Table 2:** Fuzzy rules based on features 1 and 2.

Input 1 (Feature 1)	Input 2 (Feature 2)	Output of FIS
L	L	L
L	H	M
H	L	M
H	H	H

**Table 3:** IVs obtained with four combinations.

Feature 1	Feature 2	IV	
		MIN Rule	MAX Rule
0.80(L)	0.00(L)	0.00(L)	0.80(L)
0.80(L)	1.00(H)	0.80(M)	1.00(M)
0.20(H)	0.00(L)	0.00(M)	0.20(M)
0.20(H)	1.00(H)	0.20(H)	1.00(H)

**Table 4:** Experimental results by our method with the Caltech datasets.

Database	#TP	#FP	#FN	Precision	Recall	F-Measure
Cordova 1	1201	100	141	0.92	0.89	0.91
Cordova 2	824	230	122	0.78	0.87	0.82
Washington 1	1242	259	328	0.83	0.79	0.81
Washington 2	1611	43	299	0.97	0.84	0.90
Total	4878	632	890	0.89	0.85	0.87

**Table 5:** Experimental results by our method with the SLD datasets.

Database	#TP	#FP	#FN	Precision	Recall	F-Measure
SLD	6430	553	1493	0.92	0.81	0.86

**Table 6:** Experimental results by our method with the Road Marking datasets.

Database	#TP	#FP	#FN	Precision	Recall	F-Measure
Road marking	5128	999	640	0.84	0.89	0.86

**Table 7:** Comparative experimental results by our method and previous methods.

Criterion	Methods	Caltech Dataset				SLD	Road-Marking
		Cordova 1	Cordova 2	Washington 1	Washington 2		
Precision	Ours	0.92	0.78	0.83	0.97	0.92	0.84
	[6]	0.82	0.68	0.62	0.88	0.86	0.73
	[5]	0.11	0.17	0.1	0.12	0.11	0.1
	[4]	0.54	0.3	0.54	0.42	0.40	0.58
	[7]	0.5	0.41	0.42	0.67	0.38	0.64
	[1]	0.75	0.42	0.45	0.52	0.78	0.78
Recall	Ours	0.89	0.87	0.79	0.84	0.81	0.89
	[6]	0.85	0.72	0.72	0.83	0.78	0.82
	[5]	0.08	0.13	0.06	0.05	0.08	0.02
	[4]	0.52	0.32	0.45	0.26	0.38	0.13
	[7]	0.22	0.33	0.32	0.31	0.29	0.16
	[1]	0.45	0.57	0.46	0.46	0.44	0.49
F-measure	Ours	0.91	0.82	0.81	0.90	0.86	0.86	
	[6]	0.83	0.70	0.67	0.85	0.82	0.77	
	[5]	0.09	0.15	0.08	0.07	0.09	0.03	
	[4]	0.53	0.31	0.49	0.32	0.39	0.21	
	[7]	0.31	0.37	0.36	0.42	0.33	0.26	
	[1]	0.56	0.48	0.45	0.49	0.56	0.60	

**Table 8:** Processing time per each frame by our method (unit: milliseconds).

Database	Processing Time
Cordova 1	23.47
Cordova 2	24.02
Washington 1	29.55
Washington 2	27.33
SLD dataset	17.58
Road marking dataset	30.98
Average	24.77
